# Monitoring of bacterial community structure and growth: An alternative tool for biofilm microanalysis

**DOI:** 10.1016/j.bioflm.2020.100034

**Published:** 2020-07-31

**Authors:** Guilherme O.A. da Silva, Simone Pennafirme, Daniella da Costa Pereira, Carolina C.C. Waite, Ricardo T. Lopes, Inayá C.B. Lima, Mirian A.C. Crapez

**Affiliations:** a School of Earth and Environmental Sciences, University of Queensland, St. Lucia, QLD, Australia; b Laboratório de Microbiologia Marinha e Ecologia Bacteriana, Programa de Pós-Graduação Em Biologia Marinha e Ambientes Costeiros, Universidade Federal Fluminense, Niterói, RJ, Brazil; c Nuclear Engineering Program/COPPE, Universidade Federal Do Rio de Janeiro, Rio de Janeiro, RJ, Brazil

**Keywords:** Biofilm, Bacteria, Environmental monitoring, Microtomography, Bioremediation

## Abstract

Microorganisms, such as bacteria, tend to aggregate and grow on surfaces, secreting extracellular polymeric substances (EPS), forming biofilms. Biofilm formation is a life strategy, because through it microorganisms can create their own microhabitats. Whether for remediation of pollutants or application in the biomedical field, several methodological approaches are necessary for a more accurate analysis of the role and potential use of bacterial biofilms. The use of computerized microtomography to monitor biofilm growth appears to be an advantageous tool due to its non-destructive character and its ability to render 2D and 3D visualization of the samples. In this study, we used several techniques such as analysis of microbiological parameters and biopolymer concentrations to corroborate porosity quantified by 2D and 3D imaging. Quantification of the porosity of samples by microtomography was verified by increased enzymatic activity and, consequently, higher EPS biopolymer synthesis to form biofilm, indicating growth of the biofilm over 96 ​h. Our interdisciplinary approach provides a better understanding of biofilm growth, enabling integrated use of these techniques as an important tool in bioremediation studies of environments impacted by pollutants.

## Highlights

• Biofilm formation is a strategy by which bacteria can create their own microhabitats.
• Interdisciplinary approach provides a better understanding of biofilm growth.
• Integrated use of techniques is an important tool in bioremediation studies.
• X-ray microtomography proved to be a viable technique for monitoring biofilm growth.

## 1 Introduction

In medicine, natural science and engineering, strategies to monitor biofilm growth and behavior in response to stress have become a major challenge [[1], [2], [3], [4], [5]], and several methodological approaches are necessary to provide an accurate analysis of the role and potential use of bacterial biofilms.

In recent years, imaging techniques have become a great tool for ecological studies involving bacterial biofilms, since they allow visualization of bacterial biofilms as well as bacterial cells [[6], [7], [8]]. In addition, depending on the technique employed, other factors can be observed, such as the association of pollutants with biofilms and other substances, and the monitoring of biofilm development over time. For these reasons, imaging techniques that allow monitoring of bacterial biofilms in the environment have become extremely important tools for bioremediation studies [9]. The advent of X-ray techniques are a good alternative to visualize the bacterial biofilm, mainly since they provide 2D and 3D biofilm images and allow reconstruction of images that are faithful to biofilms found in the environment.

Biofilms are defined as aggregates of microorganisms, incorporated into a matrix of extracellular polymeric substances (EPS) produced by the microorganisms themselves, adhered to each other and/or to a surface [10]. By producing EPS, bacteria create a physically distinct habitat that provides shelter, promotes accumulation of nutrients, and alters both the physicochemical environment of the biofilm and the interactions between the organisms within it [11].

These properties make biofilms one of the most widely distributed and successful lifestyles on Earth [12]. These complex life systems have high cell densities in the environment, ranging from 10^8^ to 10^11^ ​g^−1^ wet weight cells [13], and typically comprise many species of microorganisms. They are also characterized by heterogeneity, resulting from cellular differentiation triggered by local conditions and coordinated life-cycles, including gene expression and protein synthesis at specific stages, as is typical for the growth and development of microorganisms in spatially heterogeneous ecosystems [14].

The extracellular matrix facilitates communication between microorganisms, since it ensures the proximity of different metabolically-dependent physiological groups [15]. This matrix hosts the activity of extracellular enzymes in an area close to the cells, allowing efficient use of the products of enzymatic reactions for bacterial metabolism [16]. EPS therefore play a key role in biofilm formation, mass transfer through biofilms, adsorption by biofilms of different metals and organic/inorganic compounds and, most importantly, provides biofilms with structural support (shear resistance) [7,[17], [18], [19], [20], [21]].

The imaging techniques, such as computerized microtomography, allow access to these biofilm properties and corroborate the results obtained by analytical quantification of standard microbiological parameters, providing a new way to monitor the biofilm growth in the environment. Thus, when associated with other types of analysis (e.g. enzymatic, biopolymer and cell biovolume), imaging techniques and especially non-invasive approaches may become important tools for the study of biofilm behavior in the environment and its potential role in bioremediation. The main objective of this work was to monitor the growth of a bacterial biofilm in porous media by integrating the X-ray imaging techniques with several techniques already recognized for their individual utility in microbial analysis.

## 2 Material and methods

### 2.1 Sampling

**Fig. 1:**
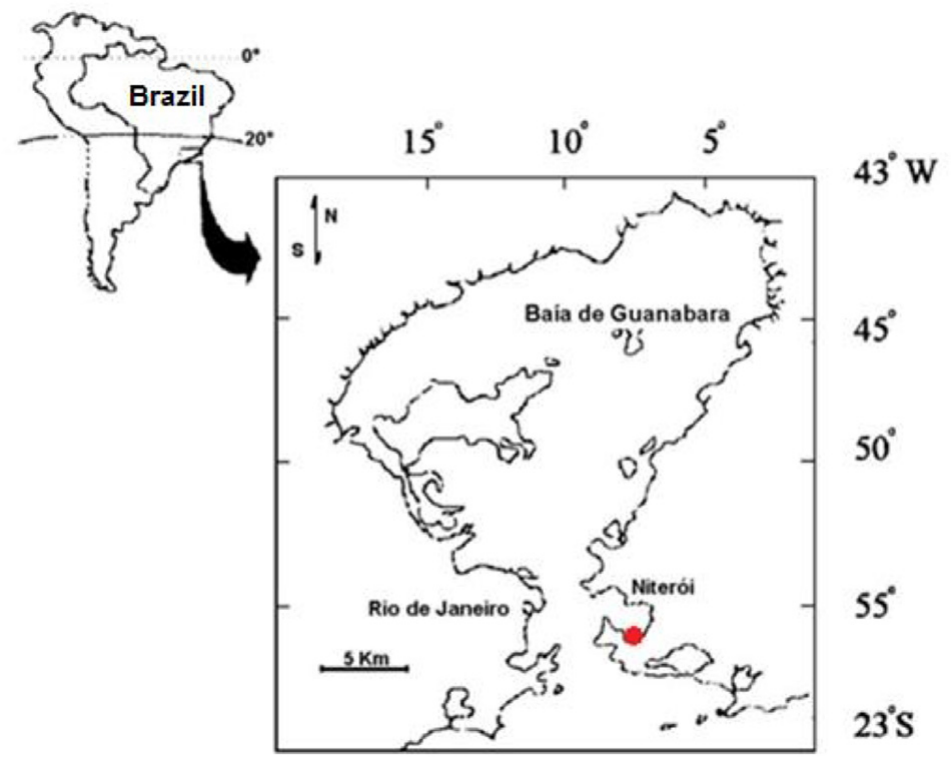
Map of the Guanabara Bay with the collection point represented in red at Jurujuba Beach, Niterói, Rio de Janeiro-Brazil. (For interpretation of the references to color in this figure legend, the reader is referred to the Web version of this article.)

The bacterial consortia were isolated from surface sediments collected in the intertidal region of Jurujuba Beach, near the entrance of Guanabara Bay, Rio de Janeiro, Brazil (22°93′88.97″S; 43°11′28.03″W). This location was selected because the region presents a history of environmental degradation over recent years and therefore we could acquire consortia resistant to the stressors present in that area (Fig. 1), such as the metals Pb, Ni, Cu, Cr, Zn and Mn [22]. The sediment was stored in a sterile plastic bottle, and packed in a cool box with ice on the way to the laboratory, where the culture medium for the bioassay was prepared (the medium specifications are in the next section).

### 2.2 Bacterial consortia maintenance and isolation

The culture medium used for isolation and maintenance of the consortia included Bacto peptone (5 ​g ​L^−1^, wt/vol) and urea (2 ​g ​L^−1^, wt/vol) as carbon and nitrogen sources, respectively, as well as 75% seawater (75% seawater ​+ ​25% deionized water). Seawater was collected at Itacoatiara beach (22°58′28.3″S 43°02′20.2″W), area without a history of pollution, located in Rio de Janeiro State, Brazil, and it was pre-filtered (Millipore®, Cellulose, 0.45 ​μm) to remove particulate material [23]. The culture medium was sterilized by autoclaving for 20 ​min at 120 ​°C [24].

An aliquot of the collected sediment was inoculated into an Erlenmeyer flask containing 250 ​ml of the culture medium and incubated in a bacteriological growth oven at 37 ​°C for 15 days before the start of the bioassay. This pre-inoculum procedure was necessary to attain a minimum biomass of bacteria, which was quantified by epifluorescence microscopy and analyzed for enzymatic activity at the time of being introduced into the bioassay (time zero).

### 2.3 Bioassay

Bioassays were adapted to allow visualization of biofilm extracted from the environment. Experiments were performed in microcosms consisting of glass cores (50 ​mm ​× ​10 ​mm), which were filled with glass microspheres (Só Esferas®) with diameter from 1.0 to 2.5 ​mm. A 150 ​μm pore size mesh was later fixed to both ends of the cores, which allowed fluid passage and retention of the microspheres throughout the bioassay. The microspheres were used as a substrate for biofilm growth, representing a porous medium such as compartmentalized coastal sediments. Each system was pre-washed with 70% ethanol (vol/vol) and the microspheres were autoclaved for 20 ​min at 120 ​°C.

**Fig. 2:**
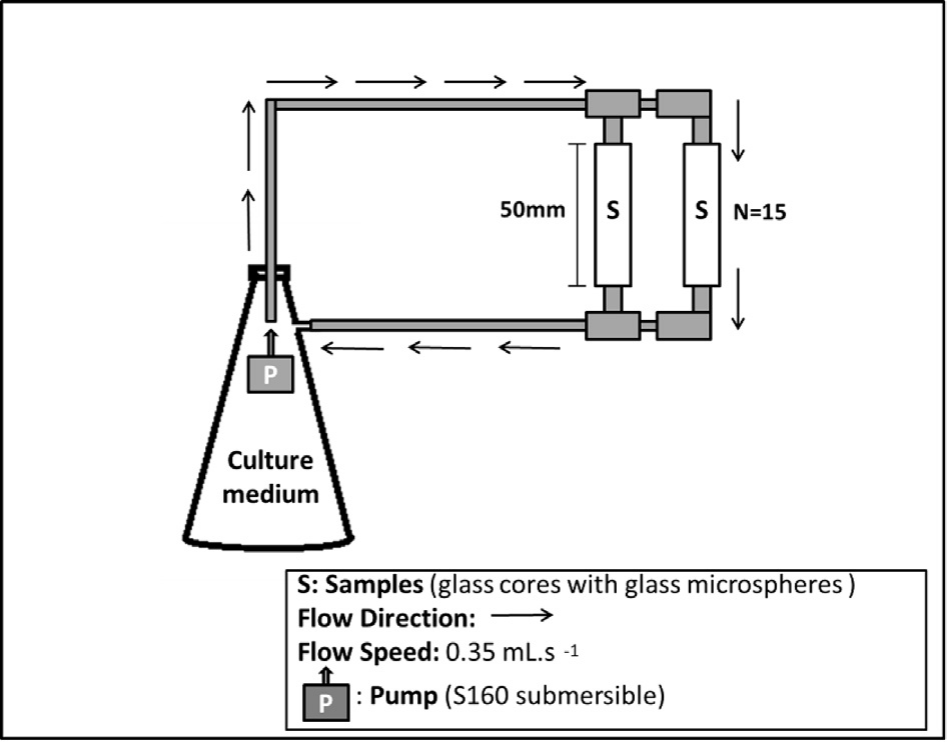
Illustrative Scheme of the bioassay. “N” represents the Number of glass cores in each experiment.

For the bioassay, the pre-inoculum was added to 3 ​L of sterile culture medium in a Kitazato flask. We inoculated 1.63 ​× ​10^9^ ​cells. cm^−3^ at the beginning of the experiment. Analyses were conducted at 0, 24, 48, 72 and 96 ​h. At each time, three tubes were removed. At the base and at the top of each tube there were connections that allowed the opening or closing for the flow to pass, thus, the removal of one tube did not affect the continuity of the experiment in the other tubes until the end of 96 ​h. 1 ​g of the tubes content (microspheres ​+ ​biofilm) was aliquoted for each triplicate of each microbiological analysis performed. All samples were analyzed in triplicates, with one control for each sample. Quantification of bacterial biomass, enzymatic activities (dehydrogenase and esterase) and microtomographic acquisition were performed at each time-point. Quantification of biopolymers was performed at time-point 0 and 96 ​h. To maintain bacterial growth throughout the experiment, an S160 submersible pump (Sarlobetter®) was used to maintain a constant flow (0.35 ​ml ​s^−1^) of the solution containing the culture medium through the cores as shown in Fig. 2 [1]. Three independent bioassays, under the same conditions and using the same protocols, were performed using the same initial culture medium for all.

### 2.4 Quantification of microbiological parameters

#### 2.4.1 Quantification of esterase enzyme activity (EST)

Quantification of EST was performed in triplicate according to Ref. [25]. The method is based on the estimate of the fluorescein produced in the sample treated with fluorescein diacetate (FDA) solution and incubated at 24 ​°C for 75 ​min on a mechanical shaker. The results were obtained using an optical spectrophotometer (Spectronic 20D®), the optical density (O.D.) was observed at a wavelength of 490 ​nm. The results are expressed in μg fluorescein. g^−1^.

#### 2.4.2 Quantification of the activity of dehydrogenase enzymes (DHA)

Quantification of DHA was performed in triplicate with the aid of an optical spectrophotometer (Spectronic 20D®) at 475 ​nm according to Ref. [26]. The method is based on the color change of INT (2[(pyodophenyl)-3-(*p*-nitrophenyl)-5-phenyl tetrazolium]), which works as an artificial electron acceptor. The product of the reaction is INT-F (iodonitrotetrazolium formazan chloride). The results are expressed as mg INT-F.mg^−1^.

#### 2.4.3 Quantification of bacterial cells

Bacterial cells number was established according to Ref. [27,28]. The samples were preserved in formaldehyde 4% (vol/vol). 75 ​μL of the chromophore Acridine Orange (100 ​μg ​ml^−1^, wt/vol) was applied to a 2 ​mL sample after serial dilution. The sample was then stained for 5 ​min before filtration with a black nuclepore membrane (Isopore Membrane, polycarbonate, Hydrophilic, 0.22 ​μm, 25 ​mm, brown, plain). Quantification was performed by epifluorescence microscopy (x1000; Axiops 50, Zeiss®, Texas Triple Red-Fluorescein-DAPI isotype), and the number of cells was estimated by calculating the total number of cells counted (sum of all fields counted), in triplicate. The results are expressed in cells. cm^−3^.

### 2.5 Quantification of biopolymers

Determination of total biopolymers (carbohydrate, lipids and proteins) was performed in triplicate. All determinations were done by the spectrophotometric method. Proteins (PRT) were analyzed by adapting the Hartree extraction [29]; with the modifications provided by Ref. [30]; using phenol compensation. For quantification, an optical spectrophotometer (Spectronic 20D®) was used at a wavelength of 650 ​nm. Bovine albumin, fraction V (Sigma), was used as a standard. Lipids (LPD) were analyzed according to Ref. [31] and tripalmitine was used as a standard. Carbohydrates (CHO) were quantified according to Ref. [32] and optimized by Ref. [33]; using glucose as standard.

### 2.6 3D monitoring of biofilm growth

#### 2.6.1 Microtomographic acquisition

A concentrated solution of BaSO_4_ (0.3 ​g ​ml^−1^, wt/vol) was added only to glass cores subjected to data acquisition, which had been removed from microcosms. The solution percolated between samples for 4 ​h prior to data acquisition. The system used for acquisition was the SKYSCAN BRUKER®, model 1173. Samples were scanned with a voltage of 70 ​kV and a current of 114 ​μA, with an aluminum filter (1.0 ​mm thickness) to minimize the beam hardening effect. Pixel size was 10 ​μm and each projection was acquired with 0.8° pitch over 360°. Due to the high resolution and the dimension of the analyzed object, 3x oversize scans were performed, for scanning the entire sample, from bottom to top, according to the system manual (Skyscan 2011a).

#### 2.6.2 Image reconstruction

Projections were reconstructed using the SkyScan NRecon® program (version 1.6.8.0), which allows reconstructions of image cross-sections from microtomography projections, especially X-ray cone beam projections [34].

#### 2.6.3 Image post-processing

Image post-processing was performed using the CT-Analyzer software (version 1.13.5.1, CTAn® - Bruker microCT), with the objective of quantifying the porosity of the samples across the 96 ​h of bioassays. All calculations were performed based on the region of interest (ROI) and thresholding (TH). All tests performed in CTAn® for sample segmentation are described in the software manuals [35,36]. Porosity was determined by differentiating the sum of the spheres and the biofilm from the voids in the sample, from which the CTAn software could calculate the porosity (%) of each sample in 3D. Total porosity was established by the ratio of total pore space to total sample volume.

All binarized objects within an ROI were analyzed together and the total pore volume, total porosity (%) and total object volume were calculated. In this work, TH represented the threshold between what is in fact the sample of interest (biofilm) and what is not (i.e. empty space in the sample). After TH determination, the sample was filtered using specific filters to improve image quality, which reduced the noise generated by data acquisition. All these parameters were determined using the CTAn® software. TH was determined using the Otsu Multilevel method (Automatic), which is based on the distribution characteristics of image tones [37].

### 2.7 Statistical treatment of data

All statistical analysis was performed using the R Core Team® software data package, Version R i386 3.3.2, Austria [38]. The *Kolmogorov-Smirnov* test was used to analyze the normality of the data and the Bartlett test for the homoscedasticity of variances [39]. As the data did not present a normal distribution and also did not present a homogeneous variance, the *Kruskal-Wallis* non-parametric test, ANOVA equivalent, was used for the analysis of microbiological parameters and porosity. The non-parametric test was applied in order to verify possible significant differences between the times in the bioassay and differences among the independent bioassays.

To observe the significant differences between the times within the bioassay, the *KruskalMC* a posteriori test, equivalent to the *Tukey* test, was applied. This test combines information from 1-way non-parametric analysis results with additional calculations to perform the non-parametric multiple comparison procedure [40]. Data were considered significant when p ​≤ ​0.05.

Spearman’s correlation coefficient non-parametric test was performed to analyze the intensity and direction of the monotonic relationship between the variables (microbiological parameters x porosity). Data were considered significant when p ​≤ ​0.05.

In the case of biopolymers, to assess whether there was a significant variation in the concentration between time 0 ​h and time 96 ​h, the *Wilcoxon t*-test was used. This test is a non-parametric method for comparing two paired samples, which replaces the *Student’s* t-test, when the data do not meet the requirements of the latter [39]. The data were considered significant when they reached p ​≤ ​0.05.

The results are illustrated by means in bar graphs, in which the average results and the standard deviation between the triplicates are presented (mean ​± ​SD), and comparative graphs in lines, in which the means are presented for each time of the bioassay.

## 3 Results

### 3.1 Analysis of microbiological parameters and biopolymers

**Fig. 3:**
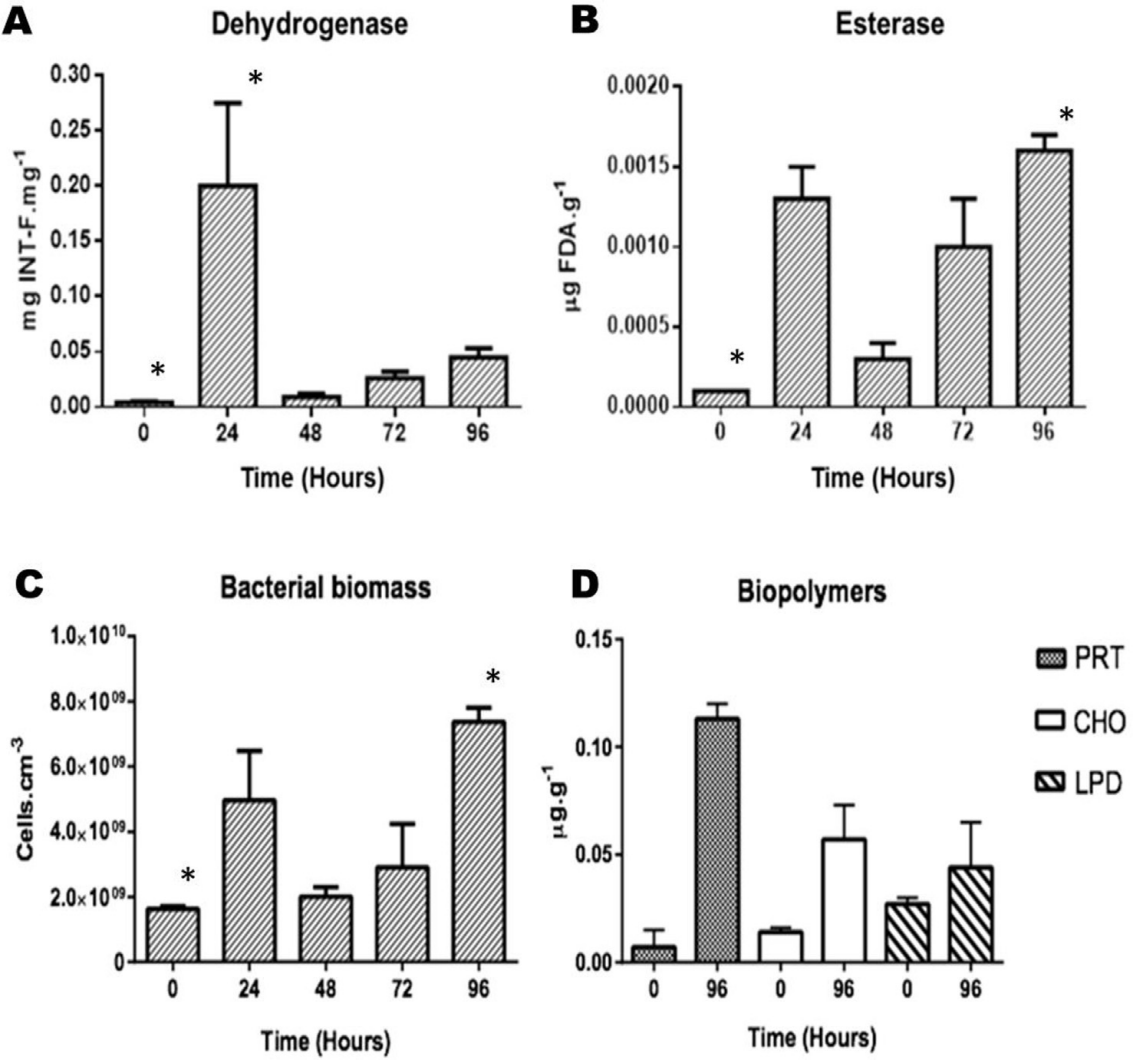
Microbiological parameters and Biopolymers analyzed during the Bioassay, with initial inoculum of 1.63 ​× ​10^9^ ​cells. mL-1. **A.** Activity of dehydrogenase enzymes (significant difference among time-points, p ​= ​0.0099); **B.** Activity of esterase enzymes (significant difference among time-points, p ​= ​0.0119); **C.** Bacterial biomass (significant difference among time-points, p ​= ​0.0148) and **D.** Analysis of biopolymers Protein (PRT), Carbohydrate (CHO) and Lipid (LPD). Results are presented as mean ​± ​SD. ∗ shows the samples that were significant different.

The results are represented by means in bar graphs with the standard deviation between the triplicates presented (mean ​± ​SD). For the bioassay (Fig. 3A–D), we provided an inoculum of 1.63 ​× ​10^9^ ​cells. cm^−3^ at time 0 ​h, representing the minimum biomass necessary for quantification of bacterial cells. During the bioassay, dehydrogenase and esterase activity exhibited the same trend as bacterial biomass, increasing exponentially in the first 24 ​h. The activity of the dehydrogenase enzymes increased from 0.004 ​± ​0.0 ​mg INT-F.mg^−1^ to 0.20 ​± ​0.07 ​mg INT-F.mg^−1^ in the first 24 ​h, and then, between 24 ​h and 48 ​h, decreased to 0.009 ​± ​0.0 ​mg INT-F.mg^−1^. Esterase activity increased from 0.0001 ​± ​0.0001 ​μg FDA. g^−1^ at the beginning of the bioassay, to 0.0013 ​± ​0.0002 ​μg FDA. g^−1^ in 24 ​h, followed by a decrease to 0.0003 ​± ​0.0001 ​μg FDA. g^−1^ in 48 ​h. Bacterial biomass increased to 4.96 ​× ​10^9^±1.52 ​× ​10^9^ ​cells. cm^−3^ in the first 24 ​h of the bioassay, and then decreased to 2 ​× ​10^9^±2.94 ​× ​10^8^ ​cells. cm^−3^ in the subsequent 24 ​h. After 48 ​h, dehydrogenase and esterase activities as well as biomass all increased again until the end of the bioassay (96 ​h), with biomass (7.37 ​× ​10^9^±4.32 ​× ​10^8^ ​cells. cm^−3^) and esterase activity (0.0016 ​± ​0.0001 ​μg FDA. g^−1^) both reaching their maximum values at the 96 ​h time-point.

The *Kruskal-Wallis* test indicated that there was a significant difference between time-points for all microbiological parameters analyzed in the bioassay. The *Kruskal-Wallis* test showed that there was a significant difference between time-points for dehydrogenase activity (p ​= ​0.0099), with the largest difference between the 0 ​h and 24 ​h time-points according to the *post hoc KruskalMC* test. A significant difference was also found for esterase activity (p ​= ​0.0119), this time the largest difference was between the 0 ​h and 96 ​h time-points *(KruskalMC* test). Bacterial biomass also differed significantly between time-points (p ​= ​0.0148) and, as for esterase activity, a *KruskalMC* test revealed this to be due to a large difference between the 0 ​h and 96 ​h time-points.

Biopolymers increased their respective concentrations at the end of the bioassay. Proteins increased from 0.007 ​± ​0.008 ​μg ​g^−1^ at the beginning of the bioassay (0 ​h) to 0.113 ​± ​0.007 ​μg ​g^−1^ at the end (96 ​h). Carbohydrates, from 0.014 ​± ​0.002 ​μg ​g^−1^ to 0.057 ​± ​0.016 ​μg ​g^−1^. And the lipids increased from 0.027 ​± ​0.003 ​μg ​g^−1^ to 0.044 ​± ​0.021 ​μg ​g^−1^. The *Wilcoxon t*-test showed no significant difference between biopolymer concentrations at time 0 ​h and 96 ​h. The *Kruskal-Wallis* test did not show any significant difference in the microbiological parameters evaluated between the 3 independent bioassays.

### 3.2 Computed microtomography analysis of porosity

**Fig. 4:**
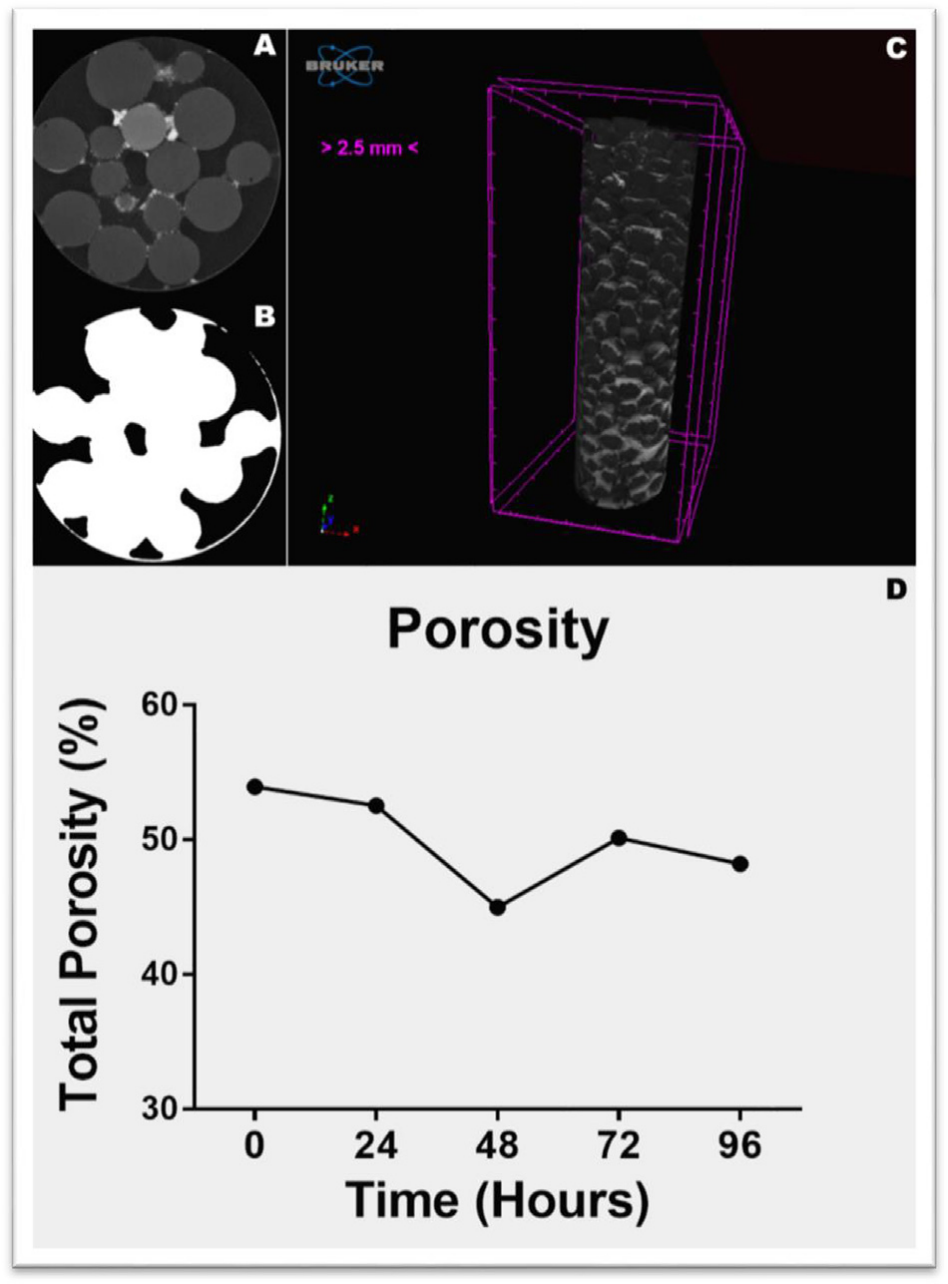
Binarization of the images and the calculation of porosity through MicroCT. A. Original image reconstructed 2D (Slice - base: h ​= ​11.5 ​mm) and B. binary (black and white) image between empty space and object; C. Sample reconstructed in 3D, non-binarized, which was used to calculate total porosity. Biofilm visible in the sample in light gray, adhered to the spheres (black); D. Variation of total porosity (%) of samples between times 0 ​h and 96 ​h.

Porosity was determined by differentiating the sum of the spheres and the biofilm (white) from the voids (black) in the sample (Fig. 4A and B), from which the CTAn software could calculate the porosity (%) of each sample in 3D (Fig. 4C). Total porosity was established by the ratio of total pore space to total sample volume (Fig. 4D).

We found that total porosity had slightly decreased in the samples after 96 ​h of the bioassay. Porosity was 53.93% at time 0 ​h and declined to 48.21% by 96 ​h (a decrease of 5.72%), with the lowest porosity recorded at the 48 ​h time-point (44.99%). A *Kruskal-Wallis* test did not evidence a significant difference in porosity between the time-points (p ​> ​0.05). However, the fact that porosity had decreased by the end of the bioassay suggests an increase in biofilm production over time. Although colonization of the biofilm was visually perceptible at the end of the bioassay, quantitative validation is necessary, such as can be provided by bacterial enzymatic analysis over time.

### 3.3 Linking microbial parameters with computerized microtomography

We found a correlation between our data on biomass and dehydrogenase activity (Fig. 3A and C) in that both increased over the 96 ​h of the bioassay. Biopolymer concentration also followed the same trend as esterase activity, increasing over the course of the bioassay (Fig. 3B and D).

**Fig. 5:**
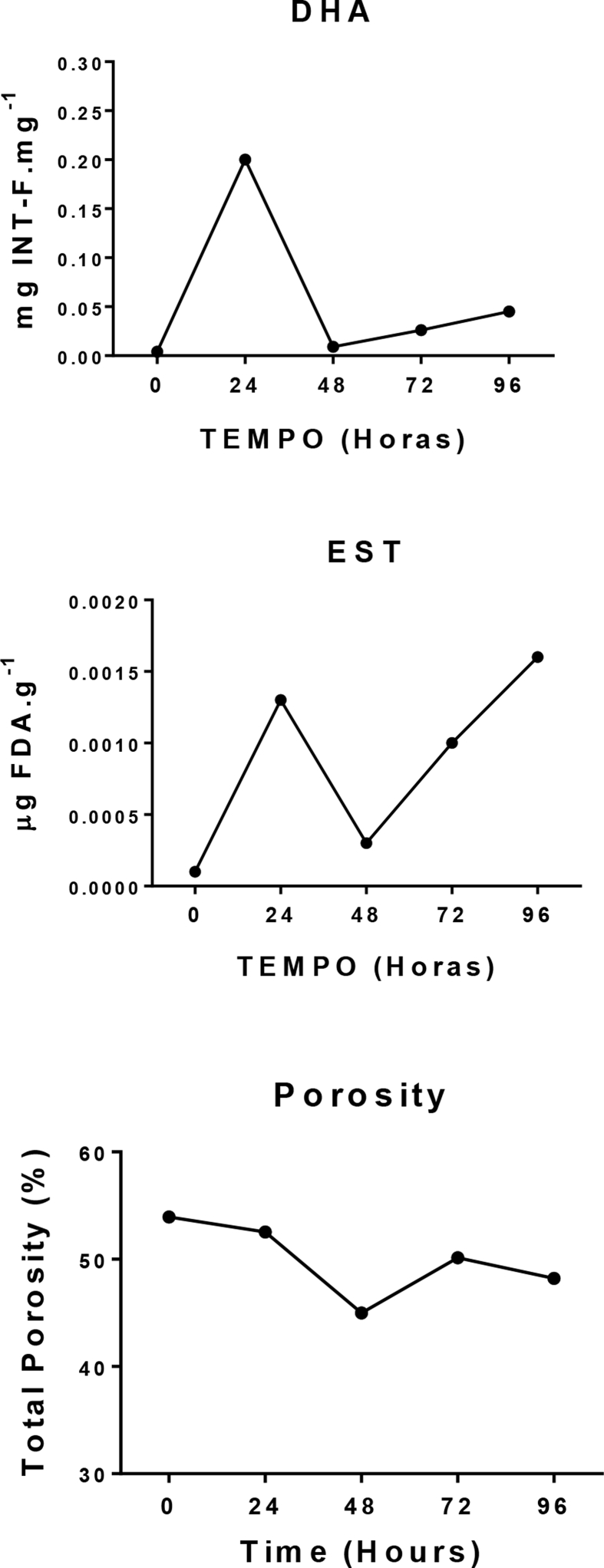
Comparative analysis between dehydrogenase (DHA) and esterase (EST) activity and porosity data generated by computerized microtomography. DHA and EST results are presented as mean, Porosity results are presented as percentage (%).

Although the graphs show an inversely proportional correlation between enzymatic activity and porosity (Fig. 5), Spearman’s correlation test showed no conclusive evidence on the significance of the association between variables, with the exception of biomass and esterase (Table 1). Increased enzymatic activity indicates an increase in EPS production and consequent biofilm formation, which could cause porosity to decrease. Thus, our quantification of enzymatic activity corroborated the results on porosity obtained through computerized microtomography.

**Table 1:** Spearman’s Correlation Test Coefficients. ∗ means significant difference.

	Porosity	Esterase	Dehydrogenase	Biomass
**Porosity**		−0.30	−0.60	−0.30
**Esterase**	−0.30		0.90	1.0∗
**Dehydrogenase**	−0.60	0.90		0.90
**Biomass**	−0.30	1.0∗	0.90	

∗Significant p-values <0.05.

## 4 Discussion

### 4.1 Microbiological parameters and biopolymers

The activities of the dehydrogenase and esterase enzymes presented the same pattern, as did bacterial biomass. Stressed communities, such as those present in Guanabara Bay, present altered energy demand, nutrients and their restoration. Energy demand among microbial communities can be measured through the activity of esterases, since these enzymes play a key role in the hydrolysis of organic matter and, consequently, in the energetic and nutrient cycles of the ecosystem [41]. Microbial cell viability and energy generation (ATP) can be assessed by the activity of dehydrogenase enzymes [25]. [41] reported that bacterial communities present in environments with high concentrations of toxic metals and organic matter exhibit increased esterase activity.

After 48 ​h of bioassay, the bacterial community began a new reproductive cycle, increasing its biomass (as shown by the enzymatic activity). Bacterial communities cycle through a sequence of phases [42], and subpopulations of biofilm cells coexist at different growth stages and, when exposed to subinhibitory concentrations of pollutants (such as metals), these cells die at different rates [43]. As a consequence, the surviving cells become less sensitive to toxic stress and the subsequent generation would likely present a genetic expression that is distinct from that of the previous generation [44]. According to Ref. [45]; this natural process of phenotypic diversification develops the resistance or tolerance of a biofilm to multiple metals, which can therefore be a useful environmental indicator.

Although the values for enzymatic activity are lower than those found for other bacterial consortia isolated from Guanabara Bay sediments [41,46,47], it was sufficient not only to sustain the bacterial community but also to increase the cellular biomass by the end of the bioassay [25]. demonstrated that the kinetics of enzymatic reactions track cell density over time, at least until the carbon source becomes a limiting factor, as observed in this bioassay.

The increased biopolymer concentrations we document here evidences synthesis of organic EPS components to sustain formation of the biofilm over the 96 ​h of bioassay. Although the increase was not statistically significant, we observed a higher amount of proteins relative to the other components at the end of 96 ​h, which has also been reported for other bacterial consortia [[48], [49], [50]]. The predominance of proteins in EPS may be due to the presence of a large amount of exoenzymes, as suggested by Refs. [51]. In addition, the higher protein content enhances hydrophobic interactions and bonding to polyvalent cations, facilitating cell aggregation and conferring greater stability to the biopolymer network [52], which are prime characteristics for the biofilm life strategy.

No significant difference was detected by the *Kruskal-Wallis* test between the microbiological results presented in the 3 independent bioassays, using the same initial inoculum for all, and also between times within a single bioassay. The standard deviation shown in the graphs demonstrates the expected variability of the replicates. In this way, the repeatability of the experiment is confirmed, demonstrating that there are no flaws even with less sampling effort.

### 4.2 Porosity analysis

Our quantification of biofilm porosity showed that there had been an increase in biofilm production by the end of the 96 ​h bioassay. Despite being efficient in other studies [53], here the porosity cannot be conclusively correlated with other analyzes, due to the lack of significant differences over time reported in this study. This lack of significant variation is probably linked to a limitation of the method and to the short duration of the bioassay, since injection of the chemical contrast agent causes detachment of weakly bonded biofilm fragments that predominate during the initial stage of growth [54]. In addition, in porous environments, space is much more limited, and biofilm growth tends to attenuate the fluid flow that supplies cells with nutrients, which makes their dispersion difficult [55].

In porous media (such as those of coastal sediments), biofilm growth can induce substantial changes in mass transport dynamics [56,57]. Variation over time of macroscopic parameters such as permeability, porosity and dispersion indicates biofilm development [1]. Thus, monitoring porosity over time using computed microtomography and corroborating the results by other techniques can be an effective ecological monitoring tool for sedimentary environments, providing a better understanding of biofilm behavior in such systems [58].

Both the spatial distribution of the biofilm and changes in porosity are important parameters to investigate the impact of biofilms on the hydrodynamics of porous media and mass transport, as well as the processes that occur during bioremediation. Our results demonstrate that computerized microtomography can provide experimental data for ratification of mathematical models of the porous media associated with biofilm growth [53,58,59].

### 4.3 Linking microbial parameters with computerized microtomography

Our comparative analysis revealed that the activity of dehydrogenase enzymes had increased by the end of the bioassay, evidencing growth of the biofilm through the energy demand (ATP). The increased biopolymer concentrations at the end of the 96 ​h bioassay indicate that there was a greater demand for the production of EPS to support the biofilm. This supposition is supported by the increased activity of esterase enzymes during the bioassay, indicating that there was a recruitment of substrate for the synthesis of EPS components.

Despite not showing significant differences, enzymatic activity was inversely proportional to the porosity of the samples, with the highest values of enzymatic activity occurring at the same time-point or before that of the lowest values of porosity. This finding can indicate that voids in the samples were colonized by biofilm throughout the bioassay [21]. suggested that energy investment under conditions of environmental stress is directed to the production of EPS. Thus, the evaluated microbiological parameters, together with our assessment of biopolymer concentrations, endorse the findings from microtomography, demonstrating how these techniques together can be used to monitor biofilm growth in porous media such as coastal sediments and soils.

Dehydrogenase and esterase activity are widely reported as indicators, respectively, of pollution and bacterial viability [7,21,41,46,47,[60], [61], [62], [63]]. According to Ref. [25]; assessment of dehydrogenase and esterase activity provides an effective means of monitoring microbial activity over time since these parameters are closely correlated with ATP content and cell density of pure and mixed microbial cultures.

Monitoring bacterial biofilms by means of integration of several correlated techniques allows better interpretation of results, leading to a better understanding of the role of biofilms in the process of bioremediation and bacterial behavioral responses to organic and inorganic pollutants. Imaging techniques such as computed microtomography enable 2D and 3D monitoring of biofilm samples, such as from contaminated soils and sediments, and quantification of their geometric and physicochemical properties.

## 5 Conclusions

Computed microtomography proved to be a viable technique for monitoring bacterial biofilm growth, with data generated by this technique being corroborated by other established methodologies. The microbiological parameters evaluated here, as well as biopolymer concentrations, served to corroborate microtomography data through their correlation with sample porosity. We also present dehydrogenase and esterase activity as good indicators, respectively, of environmental stress and bacterial viability.

Application of several integrated techniques, such as the evaluation of microbiological parameters and biopolymers and qualitative and quantitative analysis through microtomography imaging enables a better understanding of biofilm behavior and growth patterns of the same in different substrates, such as ducts and bioreactors. Thus, interdisciplinary environmental monitoring is an extremely important tool for the study and application of bioremediation techniques using bacterial biofilms in soils and coastal sediments.

## CRediT authorship contribution statement

**Guilherme O.A. da Silva:** Conceptualization, Data curation, Formal analysis, Investigation, Methodology, Validation, Writing - original draft. **Simone Pennafirme:** Conceptualization, Data curation, Formal analysis, Investigation, Methodology, Validation, Visualization, Writing - original draft. **Daniella da Costa Pereira:** Data curation. **Carolina C.C. Waite:** Data curation. **Ricardo T. Lopes:** Funding acquisition, Methodology, Resources, Software, Visualization. **Inayá C.B. Lima:** Conceptualization, Methodology, Supervision, Visualization, Writing - review & editing. **Mirian A.C. Crapez:** Conceptualization, Formal analysis, Funding acquisition, Methodology, Project administration, Resources, Supervision, Visualization, Writing - original draft.

## References

[1] Kim J., Choi H., Pachepsky Y. (2009). Biofilm morphology as related to the porous media clogging. Water Res.

[2] Rittmann B. (2007). Where are we with biofilms now? Where are we going?. Water Sci Technol.

[3] Stewart P., McFeters G., Huang C. (2000). Biofilm control by antimicrobial agents.

[4] Thormann K., Saville R., Shukla S., Spormann A. (2005). Induction of rapid detachment in *Shewanella oneidensis* mr-1 biofilms. J Bacteriol.

[5] Xavier J., Picioreanu C., Rani S., Van Loosdrecht M., Stewart P. (2005). Biofilm-control strategies based on enzymatic disruption of the extracellular polymeric substance matrix—a modelling study. Microbiology.

[6] Neu T.R., Manz B., Volke F., Dynes J.J., Hitchcock A.P., Lawrence J.R. (2010). Advanced imaging techniques for assessment of structure, composition and function in biofilm systems. FEMS Microbiol Ecol.

[7] Pennafirme S., Lima I., Bitencourt J.A., Crapez M.A.C., Lopes R.T. (2015). Microbial biofilm study by synchrotron X-ray microscopy. Radiat Phys Chem.

[8] Puyen Z.M., Villagrasa E., Maldonado J., Diestra E., Esteve I., Solé A. (2012). Biosorption of lead and copper by heavy-metal tolerant *Micrococcus luteus* DE2008. Bioresour Technol.

[9] Silva G.O.A., Pennafirme S., Lopes R.T., Lima I., Crapez M.A.C. (2017). Imaging techniques for monitoring bacterial biofilms in environmental samples – an important tool for bioremediation studies. BAOJ Microbiology.

[10] Vert M., Doi Y., Hellwich K.H., Hess M., Hodge P., Kubisa P., Rinaudo M., Schué F. (2012). Terminology for biorelated polymers and applications (IUPAC Recommendations 2012). Pure Appl Chem.

[11] Flemming H.C., Wingender J., Szewzyk U., Steinberg P., Rice S.A., Kjelleberg S. (2016). Biofilms: an emergent form of bacterial life. Nat Rev Microbiol.

[12] Stoodley P., Sauer K., Davies D.G., Costerton J.W. (2002). Biofilms as complex differentiated communities. Annu Rev Microbiol.

[13] Morgan-Sastume F., Larsen P., Nielsen J.L., Nielsen P.H. (2008). Characterization of the loosely attached fraction of activated sludge bacteria. Water Res.

[14] Singer S.W., Erickson B.K., VerBerkmoes N.C., Hwang M., Shah M.B., Hettich R.L., Banfield J.F., Thelen M.P. (2010). Posttranslational modification and sequence variation of redox-active proteins correlate with biofilm life cycle in natural microbial communities. ISME J.

[15] Wingender J., Neu T.R., Flemming H.C. (1999). Microbial extracellular substances.

[16] Bitencourt J.A.P. (2008). Influência de hidrocarbonetos aromáticos na relação trófica entre protistas de sedimento marinho e bactérias isoladas de talos algáceos. Dissertation. Programa de Pós-graduação em Biologia Marinha.

[17] Czaczyk K., Myszk K. (2007). Biosynthesis of extracellular polymeric substances (EPS) and its role in microbial biofilm formation. Pol J Environ Stud.

[18] Flemming H.C., Leis A. (2003). Sorption properties of biofilms, encyclopedia of environmental microbiology.

[19] Flemming H.C., Wingender J., Griebe T., Mayer C., Evans L.V. (2005). Physicochemical properties of biofilms. Biofilms: recent advances in their study and control.

[20] Neyens E., Baeyens J., Dewil R., De Heyder B. (2004). Advanced sludge treatment affects extracellular polymeric substances to improve activated sludge dewatering. J Hazard Mater.

[21] Waite C.C.C., Silva G.O.A., Bitencourt J.A.P., Sabadini-Santos E., Crapez M.A.C. (2016). Copper and lead removal from aqueous solutions by bacterial consortia acting as biosorbents. Mar Pollut Bull.

[22] Baptista Neto J.A., Barreto C.F., Silva M.A.M., Smith B.J., Mcallister J.J., Vilela C.G. (2013). Nearshore sedimentation as a record of landuse change and erosion: Jurujuba Sound, Niterói, SE Brazil. Ocean Coast Manag.

[23] Weber C. (1973). Biological field and laboratory methods for measuring the quality of surface waters and effluents.

[24] Madigan M.T., Martinko J.M., Parker J., Fernández M.G., Pérez M.S. (2004). Biología de los microorganismos. Brock (10th). Pearson Education.

[25] Stubberfield L.C.F., Shaw P.J.A. (1990). A comparison of tetrazolium reduction and FDA hydrolysis with other measures of microbial activity. J Microbiol Methods.

[26] Houri-Davignon C.H., Relexans J.C. (1989). Measurement of actual electrons transport system (ETS): activity in marine sediments by incubation with INT. Environ Technol Lett.

[27] Carlucci A.F., Craven D.B., Robertson K.J., Williams P.M. (1986). Surface-film microbial populations: diel amino acid metabolism, carbon utilization, and growth rates. Mar Biol.

[28] Kepner R., Pratt J.R. (1994). Use of fluorochromes for direct enumerations of total bacteria in environmental samples: past and present. Microbiol Rev.

[29] Hartree E.F. (1972). Determination of proteins: a modification of the Lowry method that give a linear photometric response. Anal Biochem.

[30] Rice D.L. (1982). The detritus nitrogen problem: new observation and perspective from organic geochemistry. Mar Ecol Prog Ser.

[31] Marsh B.J., Weinstein D.B. (1966). Simple charring method for determination of lipids. J Lipid Res.

[32] Dubois M., Gilles K., Hamilton J.K., Rebers P.A., Smith F. (1956). Colorimetric method for determination of sugars and related substances. Anal Chem.

[33] Gerchacov S.M., Hachter P.G. (1972). Improved technique for analysis of carbohydrates in sediment. Limnol Oceanogr.

[34] Machado A.C., Teles A.P., Pepin A., Bize-Forest N., Lima I., Lopes R.T. (2016). Porous media investigation before and after hydrochloric acid injection on a pre-salt carbonate coquinas sample. Appl Radiat Isot.

[35] Remy E., Thiel E. (2002). Medial axis for chamfer distances: computing look-up tables and neighbourhoods in 2D or 3D. Pattern Recogn Lett.

[36] Skyscan (2013).

[37] Otsu N. (1979). A threshold selection method from gray-level histogram. IEEE Trans Syst Man Cybern B Cybern.

[38] R Core Team (2016). R: a language and environment for statistical computing. https://www.R-project.org/.

[39] McDonald J.H. (2014). Handbook of biological statistics.

[40] Elliott A.C., Hynan L.S. (2010). A SAS® macro implementation of a multiple comparison post hoc test for a Kruskal–Wallis analysis. Comput Methods Progr Biomed.

[41] Sabadini-Santos E., Silva T.S., Lopes-Rosa T., Mendonça-Filho J.C., Santelli R.E., Crapez M.A.C. (2014). Microbial activities and bioavailable concentrations of Cu, Zn, and Pb in sediments from a tropic and eutrophicated bay. Water Air Soil Pollut.

[42] Roszak D.B., Colwell R.R. (1987). Survival strategies of bacteria in the natural environment. Microbiol Rev.

[43] Harrison J.J., Ceri H., Roper N.J., Badry E.A., Sproule K.M., Turner R.J. (2005). Persister cells mediate tolerance to metal oxyanions in *Escherichia coli*. Microbiology.

[44] Lewis K. (2007). Persister cells, dormancy and infectious disease. Nat Rev Microbiol.

[45] Harrison J.J., Ceri H., Turner R.J. (2007). Multimetal resistance and tolerance in microbial biofilms. Nat Rev Microbiol.

[46] Crapez M.A.C., Baptista Neto J.A., Bispo M.G.S. (2003). Bacterial enzymatic activity and bioavailability of heavy metals in sediments from Boa Viagem Beach, Guanabara Bay, RJ, Brazil. Anu Inst Geociênc UFRJ.

[47] Silva F.S., Santos E.S., Laut L.L.M., Sanchez-Nuñes M.L., Fonseca E.M., Baptista-Neto J.A., Mendonça-Filho J.G., Crapez M.A.C. (2010). Geomicrobiology and biochemical composition of two sediment cores from Jurujuba Sound - Guanabara Bay – SE Brazil. Anu Inst Geociênc UFRJ.

[48] Li X.Y., Yang S.F. (2007). Influence of loosely bound extracellular polymeric substances (EPS) on the flocculation, sedimentation and dewaterability of activated sludge. Water Res.

[49] More T.T., Yan S., Tyagi R.D., Surampalli R.Y. (2015). Biopolymers production by mixed culture and their applications in water and wastewater treatment. Water Environ Res.

[50] Subramanian S.B., Yan S., Tyagi R.D., Surampalli R.Y. (2010). Extracellular polymeric substances (EPS) producing bacterial strains of municipal wastewater sludge: isolation, molecular identification, EPS characterization and performance for sludge settling and dewatering. Water Res.

[51] Frølund B., Griebe T., Nielsen P.H. (1995). Enzymatic activity in the activated-sludge floc matrix. Appl Microbiol Biotechnol.

[52] Xu C., Zhang S., Chuang C.Y., Miller E.J., Schwehr K.A., Santschi P.H. (2011). Chemical composition and relative hydrophobicity of microbial exopolymeric substances (EPS) isolated by anion exchange chromatography and their actinide-binding affinities. Mar Chem.

[53] Peszynska M., Trykozko A., Iltis G., Schlueter S., Wildenschild D. (2016). Biofilm growth in porous media: experiments, computational modeling at the porescale, and upscaling. Adv Water Resour.

[54] Carrel M., Beltran M.A., Morales V.L., Derlon N., Morgenroth E., Kaufmann R., Holzner M. (2017). Biofilm imaging in porous media by laboratory X-Ray tomography: combining a non-destructive contrast agent with propagation-based phase-contrast imaging tools. PLoS One.

[55] Coyte K.Z., Tabuteau H., Gaffney E.A., Foster K.R., Durham W.M. (2016). Microbial competition in porous environments can select against rapid biofilm growth. Proc Natl Acad Sci Unit States Am.

[56] Stoodley P., Dodds I., De Beer D., Scott D., Boyle J. (2005). Flowing biofilms as a transport mechanism for biomass through porous media under laminar and turbulent conditions in a laboratory reactor system. Biofouling.

[57] Shafahi M., Vafai K. (2009). Biofilm affected characteristics of porous structures. Int J Heat Mass Tran.

[58] Iltis G.C., Armstrong R.T., Jansik D.P., Wood B.D., Wildenschild D. (2011). Imaging biofilm architecture within porous media using synchrotron-based X-ray computed microtomography. Water Resour Res.

[59] Roscoat S.R., Martins J.M.F., Séchet P., Vince E., Latil P., Geindreau C. (2014). Application of Synchrotron X-ray microtomography for visualizing bacterial biofilms 3D microstructure in porous media. Biotechnol Bioeng.

[60] Fontana L.F., Filho J.G.M., Netto A.D.P., Sabadini-Santos E., Figueiredo A.G., Crapez M.A.C. (2010). Geomicrobiology of cores from suruí mangrove – Guanabara bay – Brazil. Mar Pollut Bull.

[61] Kenarova A., Radeva G., Traykov I., Boteva S. (2014). Community level physiological profiles of bacterial communities inhabiting uranium mining impacted sites. Ecotoxicol Environ Saf.

[62] Kumar S., Chaudhuri S., Maiti S.K. (2013). Soil dehydrogenase enzyme activity in natural and mine soil - a Review. Middle East J Sci Res.

[63] Wolinska A., Rekosz-Burlaga H., Goryluk-Salmonowicz A., Błaszczyk M., Stępniewska Z. (2015). Bacterial abundance and dehydrogenase activity in selected agricultural soils from Lublin Region. Pol J Environ Stud.

